# Putative variants, genetic diversity and population structure among Soybean cultivars bred at different ages in Huang-Huai-Hai region

**DOI:** 10.1038/s41598-022-06447-6

**Published:** 2022-02-11

**Authors:** Jialin Liu, Huimin Xie, Ting Lin, Congxiao Tie, Huolin Luo, Boyun Yang, Dongjin Xiong

**Affiliations:** College of Life Science, Nanchang University, Key Laboratory of Plant Resources in Jiangxi Province, Nanchang, China

**Keywords:** Genetics, Plant breeding

## Abstract

Soybean cultivars bred in the Huang-Huai-Hai region (HR) are rich in pedigree information. To date, few reports have exposed the genetic variants, population structure and genetic diversity of cultivars in this region by making use of genome-wide resequencing data. To depict genetic variation, population structure and composition characteristics of genetic diversity, a sample of soybean population composed all by cultivars was constructed. We re-sequenced 181 soybean cultivar genomes with an average depth of 10.38×. In total, 11,185,589 single nucleotide polymorphisms (SNPs) and 2,520,208 insertion-deletions (InDels) were identified on all 20 chromosomes. A considerable number of putative variants existed in important genome regions that may have an incalculable influence on genes, which participated in momentous biological processes. All 181 varieties were divided into five subpopulations according to their breeding years, SA (1963–1980), SB (1983–1988), SC (1991–2000), SD (2001–2011), SE (2012–2017). PCA and population structure figured out that there was no obvious grouping trend. The LD semi-decay distances of sub-population D and E were 182 kb, and 227 kb, respectively. Sub-population A (SA) had the highest value of nucleotide polymorphism (π). With the passage of time, the nucleotide polymorphism of SB and SC decreased gradually, however that of SD and SE, opposite to SB and SC, gave a rapid up-climbing trend, which meant a sharp increase in genetic diversity during the latest 20 years, hinting that breeders may have different breeding goals in different breeding periods in HR. Analysis of the PIC statistics exhibited very similar results with π. The current study is to analyze the genetic variants and characterize the structure and genetic diversity of soybean cultivars bred in different decades in HR, and to provide a theoretical reference for other identical studies.

## Introduction

Soybean [*Glycine max* (L) Merrill], which originated from China, is an important economic and oil crop^1,2^, and it has been cultivated for about 5000 years^3,4^. Soybean cultivars play a crucial role in soybean production, act as the most precious germplasm resources^5^ and are widely known as domesticated from wild materials through artificial directional selection and variation accumulation^6,7^. Huang-Huai-Hai region (HR) is the second main soybean-producing area in China only after Northeast China^8,9^. In recent years, HR soybean planting area has been stabilized at 2 million hectares, accounting for about 30% of China's total soybean planting area. At the same time, HR is also one of the areas where soybean breeding started very early^10^. From the earlier cultivated "Jinda 332", a large number of cultivated varieties suitable for landrace growth have been bred^8^. According to available statistics, a total of 550 varieties were bred in the region from 1986 to 2016 alone. In addition, HR soybean varieties are rich in pedigree information and have a long time span^11^. Consequently, they are ideal materials for studying the transmission and genetic relationship of excellent allelic variations in pedigree^12,13^. All of the above information highlights the vital importance of the HR region in China. The study on the population genomic variation, genetic diversity and population genetic structure of soybean cultivars in HR can provide reference for the selection of soybean breeding parents and broadening the genetic basis of soybean breeding.

With the rapid development of high-throughput sequencing technology^14–16^, the assembly of high-quality reference genomes^17,18^ and the advent of advanced bioinformatics methods^19–21^, we can study the genetic variations at the whole genome level quickly and accurately. Then the possible genomic regions of variations and their effects on genes can be inferred by means of variation annotation. Zhou et al*.*^22^ sequenced a total of 302 soybean accessions including wild, landrace, cultivated soybean germplasm and some foreign germplasm, and a total of 9,790,744 SNPs and 976,799 InDels were detected. It was found that the occurrence of variations led to the differentiation of genes in different geographical regions, which determined the model of regional adaptability of agronomic traits. Maldonado et al*.*^23^ sequenced 28 soybean cultivars of Brazilian and analyzed in detail the possible effects of SNPs and InDels with non-synonymous mutations on the function of the corresponding genes, which suggested that once the effects of these mutations on gene functions were confirmed, then the adaptation mechanism of soybean to tropical conditions in Brazil could be explained. Kim et al*.*^24^ carried out high-depth (> 13×) whole-genome (WGS) analysis of 418 domesticated soybean cultivars, 345 wild soybean materials and 18 natural hybrid varieties. The whole genome variation map of soybean was obtained, and 10.6 million SNPs and 1.4 million InDels were identified. Further analysis found that the emergence of a large number of putative variants led to changes in gene function, and the author concluded that harmful mutations were the genetic basis of crop inbreeding decline and heterosis.

The rapid development of high-throughput sequencing technology not only enables us to obtain the information of genome-wide variation quickly and accurately, but also greatly facilitates the analysis of downstream population genetics. Now, we can study the population structure and genetic diversity at the genome-wide level to reveal the impact of genetic improvement on germplasm populations. Numerous previous studies have conducted in-depth analysis of soybean population genetic diversity, population structure and genomic LD changing patterns from the perspective of wild, landrace and cultivated or even different types and different ecological region materials, and the information obtained has been well used to guide breeding practice, meanwhile greatly promoted breeder's understanding of the genome of germplasm resources^22,25–27^. However, a practical problem that we should pay attention to is that the innovation of germplasm resources is regional adaptability, and our understanding of genetic diversity of germplasm resources should be multi-angle and multi-level. In order to apply the research results of genetic diversity of germplasm resources to breeding guidance in a better way, we must analyze the genetic diversity of soybean germplasm resources from different perspectives. So far, few relative studies have been conducted to analyze the genetic diversity of cultivated soybean populations in different decades in a single geographical region, which was thought to be very necessary^28^. Previous studies have pointed out that 80% of the cultivars bred in the HR were cultivated by cross breeding of high-yield varieties, and some breeders worried that such a "high by high" cross will reduce genetic diversity^9,29^, which was the vital factor that stimulate our interest in the study of population genetic diversity of bred varieties in HR.

A large number of excellent varieties have been bred in HR in the past few decades. In line with the idea of analyzing the genetic information of germplasm resources in HR, the main purpose of the current study is to make utilization of the sequencing information of 181 soybean cultivars in HR, which were bred in the period of 1963 to 2017, and to do the researches as following: (1) figure out a large amount of important genes that affected by putative genetic variants; (2) clarify population structure and LD variation patterns of sub-populations in each breeding period in HR; (3) portray the genetic diversity of sub-populations in different ages.

## Results

### Sequencing and variation

**Figure 1:**
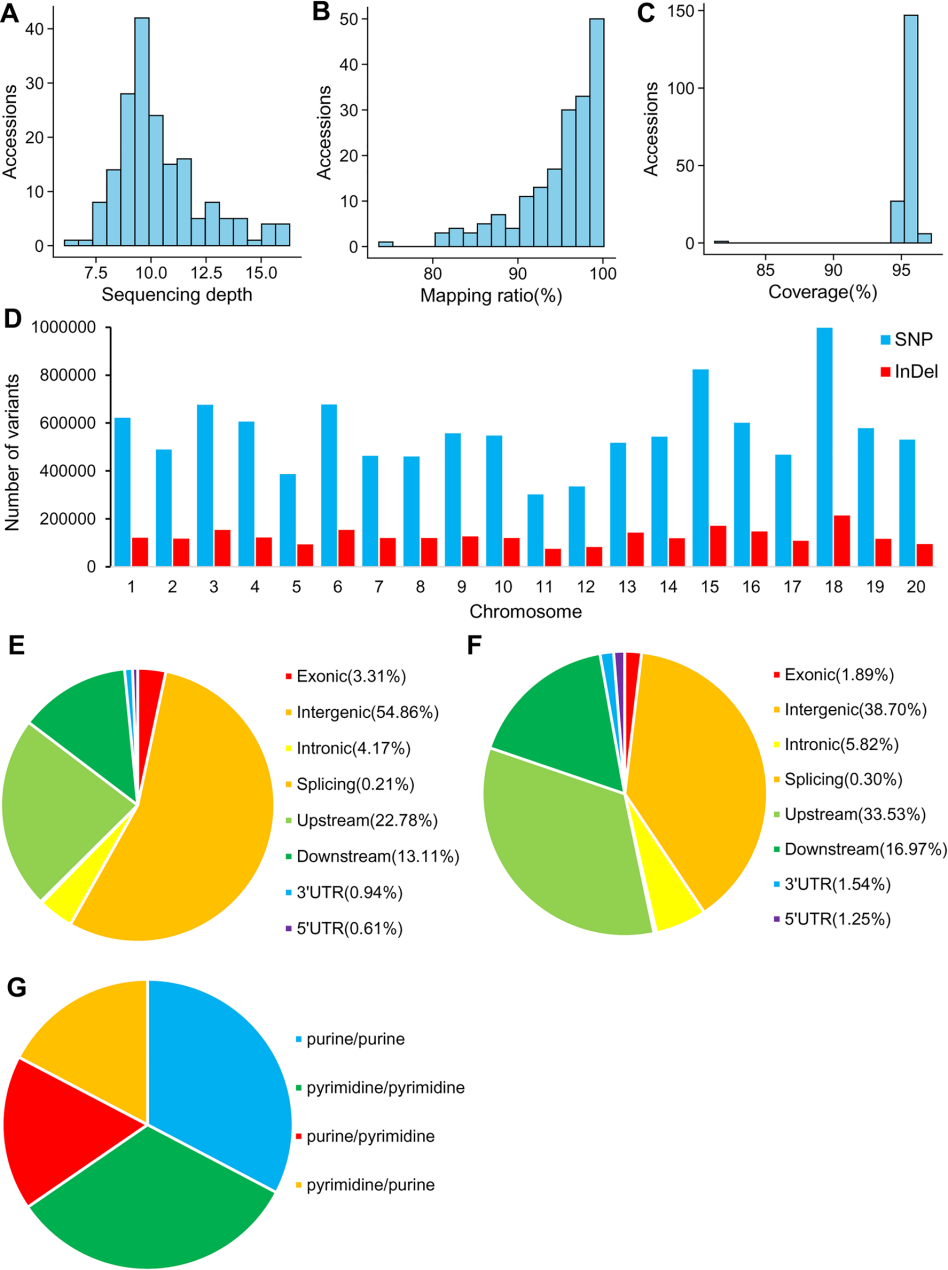
Summary of sequencing, SNPs and InDels information on the genome. (**A**) Frequency distribution histogram of sequencing depth of 181 cultivars in HR. (**B**) Frequency distribution histogram of mapping ratio of 181 cultivars in HR. (**C**) Frequency distribution histogram of coverage ratio of 181 cultivars in HR. (**D**) SNP and InDel counts on every chromosome. (**E**) Percentage of SNPs on each soybean genome region. (**F**) Percentage of InDels on each soybean genome region. (**G**) Numbers of transition/transversion mutations. Note that the plots were generated using Microsoft Excel 2016.

We re-sequenced 181 soybean cultivars in Huang-Huai-Hai region (HR) and produced approximately 15.2 billion 100 bp pair-ended reads. The sample sequencing depth ranged from 6.64 ~ 16.22 fold of the reference genome, and the average sequencing depth was 10.38× (Supplementary Table 1, Fig. 1A). Approximately 74.96 ~ 99.78% of the reads of each accession can be mapped onto the reference genome, with a mean mapping ratio and coverage rate of 94.87%, 95.48%, respectively (Supplementary Table 1, Fig. 1B,C), indicating that the sequencing covered most of the reference genome. After comparing with the reference genome, we identified 11,185,589 SNPs in all 181-soybean materials, which was more than the number of mutations in other studies^22,23,41^. These SNPs were evenly distributed on all chromosomes, among which the number of SNPs on chromosome 18 was the highest and so was the density, while chromosome 15 took the second place (Supplementary Table 2, Supplementary Fig. 1, Fig. 1D). The genome transitions/transversions ratio of 181 materials in HR was 1.89 (ts/tv ratio) (Fig. 1G). After annotation, a total of 6,136,859 SNPs were detected in the intergenic region. In the gene region, we detected 370,289 SNPs in exons and 172,761 SNPs in UTR region (Supplementary Table 3, Fig. 1E). The synonymous and non-synonymous mutation rate of 181 materials in HR was 1.60.

In this study, we detected a total of 2,520,208 InDel markers through mutation detection, which was more than the number of mutations detected in other studies^22,23,41^. For InDel markers, their distribution on chromosomes was basically consistent with the previous description of SNPs (Supplementary Table 2, Supplementary Fig. 1, Fig. 1D). There were about 975,395 InDels in intergenic region, 146,578 in intron region, 70,379 InDels in UTR region and 47,724 InDels in exon region (Supplementary Table 3, Fig. 1F).

### Variations in the genome

The variations found in HR cultivars’ genome led to a large number of codon changes in important gene regions. In this part of the present study, by comparing with the reference genome, we found a large number of genes affected by putative variations.

We found that 50,365 loci were mutated in all cultivars in HR, of which 30,624 were SNPs, and 19,741 were InDels.

A total of 3240 SNPs were identified in 1314 important gene regions in all accessions. According to the enrichment analysis of SoyBase (http://soybase.org), these 1314 genes were widely involved in biological processes such as metabolic process, cellular process, reproductive process, obsolete GTP catabolic process, regulation and so on (Supplementary Fig. 2). In addition, some genes were annotated to involve in the gene localization process, which we thought might account for the adaption of varieties to the environment of HR.

**Figure 2:**
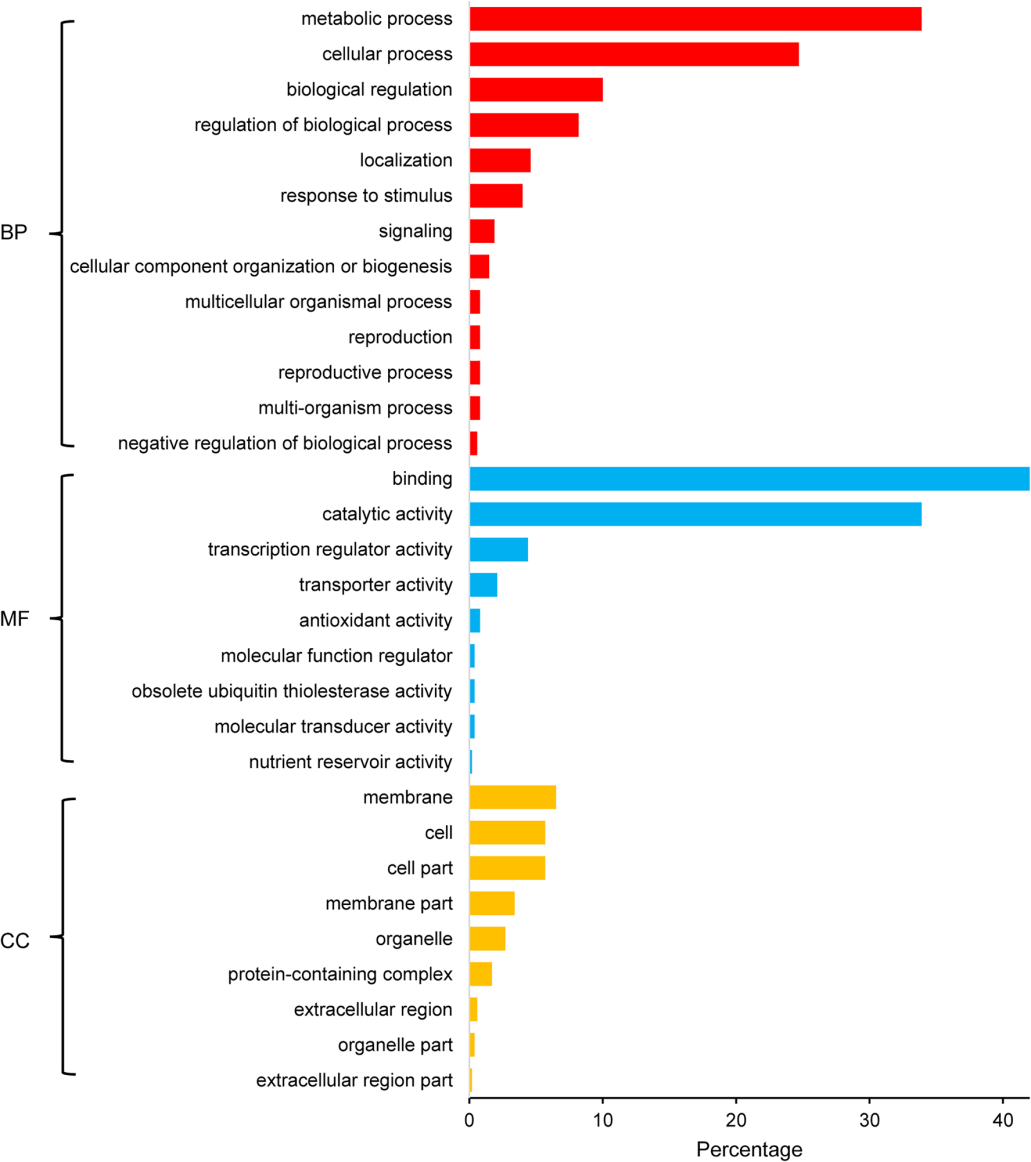
Gene ontology annotation plot for 1314 genes containing SNPs which were mutated in all varieties in HR. *BP* biological process, *MF* molecular function, *CC* cellular component. The x axis is the percentage of genes under a GO term to the total number of annotated genes. Note that the plot was generated using Microsoft Excel 2016.

Among these mutations, 1368 SNPs were located in exons of genes, and 473 were synonymous mutations, while the remaining 895 SNPs existing in 522 genes were non-synonymous mutations (Fig. 2). Among them, two SNPs were annotated as “initiator_codon_variant”, and may cause the start codon loss of *Glyma.01G016800* and *Glyma.08G024100*. In addition, 14 SNPs led to the early appearance of premature stop codons of 13 genes, leading to shortened polypeptides of *Glyma.01G009500*, *Glyma.02G287400*, *Glyma.03G034400*, *Glyma.03G081100*, *Glyma.03G173800*, *Glyma.08G228400*, *Glyma.12G181000*, *Glyma.14G209700*, *Glyma.15G166300*, *Glyma.16G121600*, *Glyma.18G279800*, *Glyma.20G015100* and *Glyma.20G240900*, and these 13 genes were mainly participated in metabolism, response to external stimuli and some regulation processes. Moreover, another four SNPs were likely to lead to the loss of termination codons of *Glyma.03G049800*, *Glyma.18G100200* (binding), *Glyma.18G161900* (binding) and *Glyma.20G135500*, but unfortunately, the biological process annotation information of *Glyma.03G049800*, *Glyma.20G135500* was not included in SoyBase (http://soybase.org) (Table 1).

**Table 1:** Summary of the most relevant results from the GO enrichment analysis of the genes affected by non-synonymous SNPs.

Description	Genes	Sequence ontology	Non-synonymous SNPs
Response to stimulus	Glyma.03g081100	stop_gained	1
Metabolic process	Glyma.01g009500	stop_gained	2
	Glyma.03g081100	stop_gained	1
	Glyma.14g209700	stop_gained	1
	Glyma.20g015100	stop_gained	1
	Glyma.20g240900	stop_gained	1
Cellular process	Glyma.03g081100	stop_gained	1
	Glyma.20g240900	stop_gained	1
Biological regulation	Glyma.03g081100	stop_gained	1
Cellular component organization or biogenesis Binding	Glyma.03g081100	stop_gained	1
	Glyma.03g034400	stop_gained	1
	Glyma.03g173800	stop_gained	1
	Glyma.15g166300	stop_gained	1
	Glyma.18g100200	stop_lost	1
	Glyma.18g161900	stop_lost	1
Catalytic activity	Glyma.01g009500	stop_gained	2
	Glyma.03g081100	stop_gained	1
	Glyma.20g015100	stop_gained	1
	Glyma.20g240900	stop_gained	1
Membrane	Glyma.20g240900	stop_gained	1

**Figure 3:**
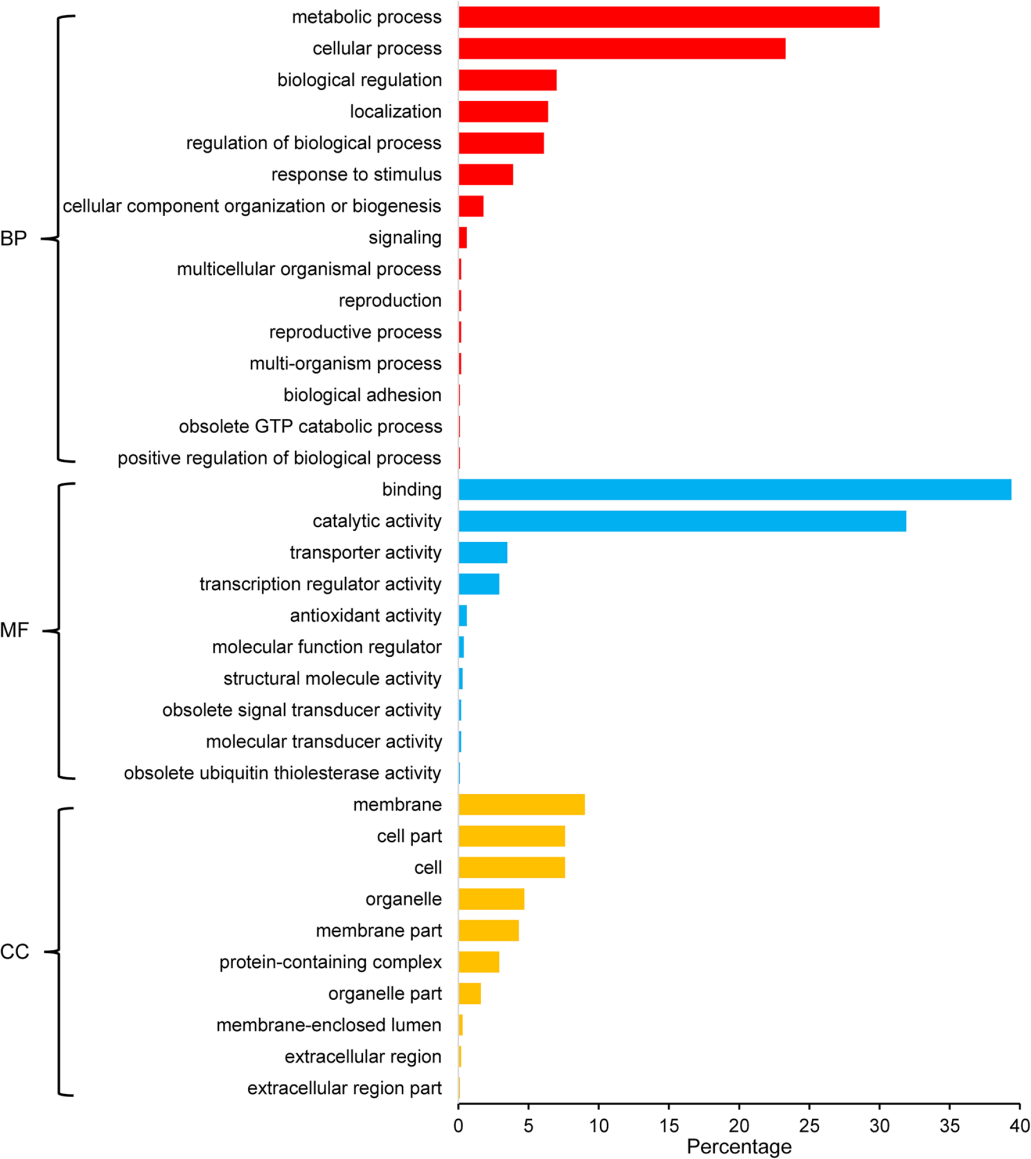
Gene ontology annotation plot for 1241 genes containing InDels which were mutated in all varieties in HR. *BP* biological process, *MF* molecular function, *CC* cellular component. The x axis is the percentage of genes under a GO term to the total number of annotated genes. Note that the plot was generated using Microsoft Excel 2016.

A non-synonymous mutation analysis for the InDel was also carried out, which identified InDels showing up in important gene regions in HR genome (Supplementary Fig. 3). 2205 InDels in important gene regions were identified to have different degree of influence on 1241 genes (Fig. 3), of which, 660 InDels were exonic variants. We annotated the 660 InDel variants and found that twelve of them were “conservative inframe deletion” variants, which can lead to the deletion of at least one complete codon in the CDS of *Glyma.10G188800*, *Glyma.14G088700* and *Glyma.15G074100*. This change was likely to change the protein coding products of the corresponding genes. Contrary to the mutation of codon deletion, we found nine “conservative inframe insertion” InDel variants, which were thought to lead to the insertion of at least one codon into CDS regions of *Glyma.03G248400*, *Glyma.04G086400*, *Glyma.07G140200*, *Glyma.07G230200*, *Glyma.15G070700*, *Glyma.16G001000*, *Glyma.16G066200*, *Glyma.19G152500* and *Glyma.19G179300*. In addition, we found that 622 InDels were “frameshift” type variants, which might cause at least one base to be deleted or inserted downstream of the mutation site, in hence may lead to changes in subsequent amino acid coding, and among which three frameshift mutations may lead to the loss of the starting codon of *Glyma.01G013200*, *Glyma.02G022800* and *Glyma.16G132100*. Moreover, another seven frameshift mutations can result in the loss of stop codons of *Glyma.04G030100*, *Glyma.04G110000*, *Glyma.07G078000*, *Glyma.09G146200*, *Glyma.11G097000*, *Glyma.15G187700* and *Glyma.16G080500*. Furthermore, there were three InDels may have the effect that can lead to the early appearance of stop codons of *Glyma.09G135600*, *Glyma.15G234300* and *Glyma.09G278400*. Surprisingly, all the genes mentioned upward in this part were mainly involved in the metabolic process and had catalytic activity and binding function and only a small range of them participated in cellular process and localization. Notably, we also found an InDel, which was annotated as a “bidirectional gene fusion” type mutation, and its production can fuse *Glyma.13G168900* and *Glyma.13G169000* together. In addition, after GO annotation, we found that *Glyma.13G168900* was involved in photosystem I assembly process, but the *Glyma.13G169000* had no corresponding annotation message in SoyBase (http://soybase.org) (Table 2).

**Table 2:** Summary of the most relevant results from the GO enrichment analysis of the genes affected by non-synonymous InDels.

Description	Genes	Sequence ontology	Non-synonymous InDels
Metabolic process	Glyma.01g013200	frameshift_variant&start_lost	1
	Glyma.04g030100	frameshift_variant&stop_lost	1
	Glyma.04g086400	conservative_inframe_insertion	1
	Glyma.15g187700	frameshift_variant&stop_lost	1
	Glyma.15g234300	frameshift_variant&stop_gained	1
Cellular process	Glyma.15g187700	frameshift_variant&stop_lost	1
Localization	Glyma.16g080500	frameshift_variant&stop_lost	1
Catalytic activity	Glyma.01g013200	frameshift_variant&start_lost	1
	Glyma.04g030100	frameshift_variant&start_lost	1
	Glyma.04g086400	conservative_inframe_insertion	1
	Glyma.15g187700	frameshift_variant&stop_lost	1
	Glyma.15g234300	frameshift_variant&stop_gained	1
	Glyma.16g066200	conservative_inframe_insertion	1
	Glyma.19g179300	conservative_inframe_insertion	1
Binding	Glyma.04g030100	frameshift_variant&start_lost	1
	Glyma.07g078000	frameshift_variant&stop_lost	1
	Glyma.07g140200	conservative_inframe_insertion	1
	Glyma.09g278400	frameshift_variant&stop_gained	1
	Glyma.14g088700	conservative_inframe_deletion	1
	Glyma.15g187700	frameshift_variant&stop_lost	1
	Glyma.16g066200	conservative_inframe_insertion	1
	Glyma.16g080500	frameshift_variant&stop_lost	1
	Glyma.19g179300	conservative_inframe_insertion	1

### Population structure analysis

**Figure 4:**
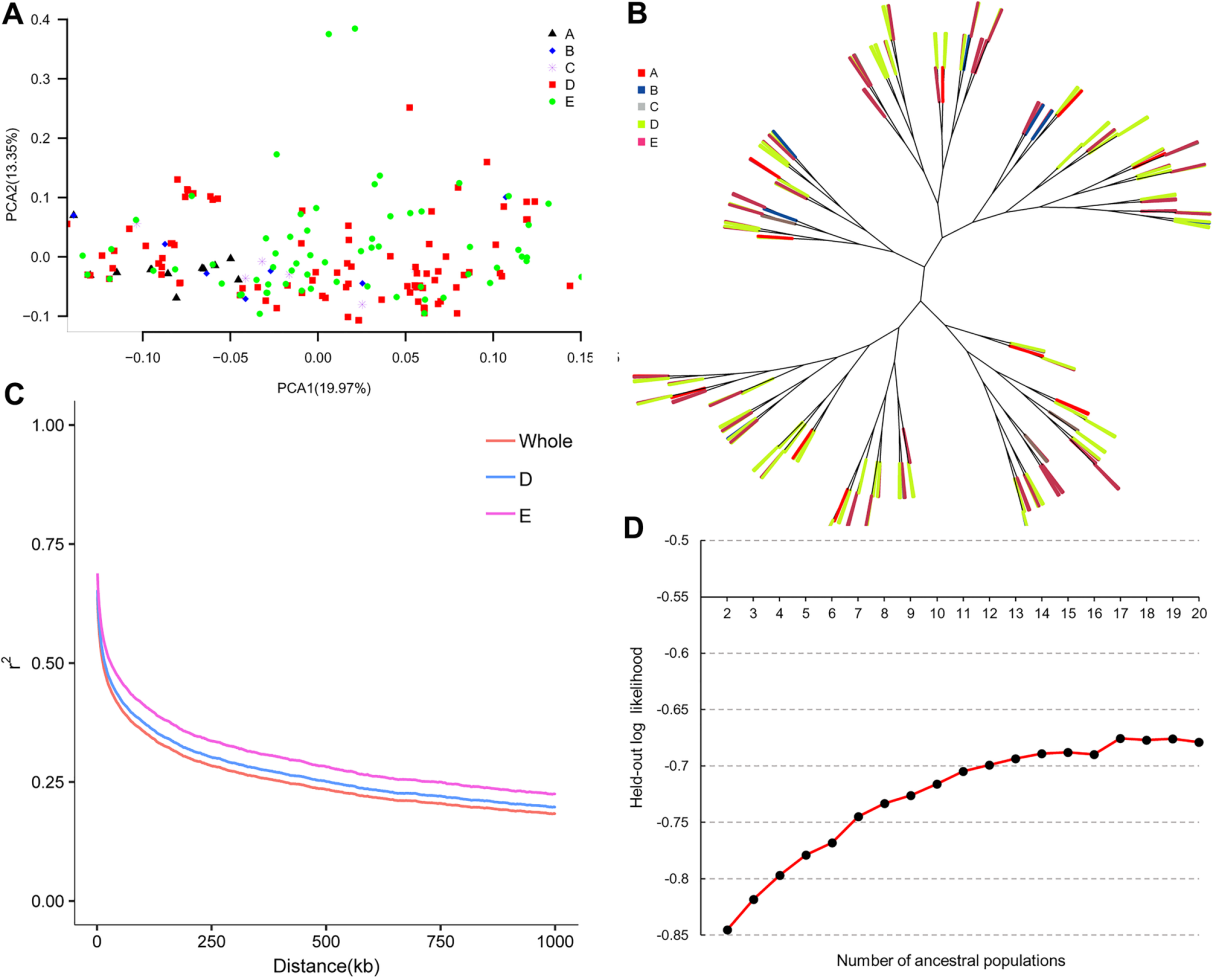
Population structure analysis. (**A**) Principal component analysis chart (PCA) of soybean cultivars in the HR. (**B**) Neighbor-joining (NJ) tree. (**C**) LD decay of SD, SE and the entire group. (**D**) Predictive log-likelihood as a function of the number of ancestral populations on the HR cultivated soybean population. Note that plot A and C were generated by self-written R scripts with R language version 4.02 (http://www.R-project.org), plot B was generated by ITOL (https://itol.embl.de/) and plot D was generated using Microsoft Excel 2016.

Population structure was completed by using the core SNP set as described in the method part. First, we conducted a principal component analysis (PCA) analysis to reveal the population structure. PCA1 and PCA2 explained the variation of 19.97% and 13.35% of the population respectively (Fig. 4A) but basing on the first two principal components (PCA1 and PCA2), the HR soybean population did not exhibit obvious subgroup clustering. The materials of SA, SB, SC, SD and SE all showed a distributed distribution. However, all the varieties of SA gathered in a relatively small range, and as the breeding period of each sub-population processing, the variation range of sub-populations gradually expanded and SE got the largest range of variation.

Next, the genetic relationship matrix among all the samples was computed by using the core SNP data set, and the NJ tree between samples was drawn (Fig. 4B), which gave a similar result with PCA. NJ tree showed that although the population of cultivars bred in the HR showed a certain tendency of clustering, the materials of sub-populations in each breeding period did not present obvious clustering characteristics. Furthermore, a cluster analysis was carried out on the present population by using Terastructure (Fig. 4D), and it was found that the value of the validation likelihood was in a climbing state until a peak occurred at k = 17. As the value of k increasing, the validation likelihood remained unchanged, and this result indicated that there might be more than 17 ancestral populations in the breeding populations in the HR.

### Linkage disequilibrium decay analysis

In this part, r^2^ was used to reflect the degree of LD of sub-populations. The half-decay distance (physical distance when r^2^ decays to half of the maximum value) of the entire HR population was 160 kb, and 182 kb, and 227 kb for sub-population D and E, respectively. The number of individuals in the three sub-populations A, B and C is relatively small. Considering that the use of a small number of samples to estimate the genomic LD may produce large errors, we had not evaluated the genomic LD of these three sub-populations (Fig. 4C).

### Genetic diversity among sub-populations

In this section, we calculate the nucleotide diversity (π) (Table 3, Supplementary Fig. 4) and the polymorphic information content (PIC) (Table 3) of sub-populations at each breeding stage in order to understand the effect of artificial selection within sub-populations on total genetic diversity. The results of π showed that among all the five sub-populations, SA had the highest nucleotide diversity (1.54 × 10^–3^), which was significantly higher than that in the later breeding stage sub-populations, while SB (1.27 × 10^–3^) and SC (1.24 × 10^–3^) got the lowest. With the passage of breeding period, the nucleotide polymorphism of SD (1.41 × 10^–3^) and SE (1.38 × 10^–3^) gradually increased, and significantly higher than that of SB and SC. The results of PIC analysis were consistent with π. SA had the highest polymorphic information content (0.242), followed by SD (0.234), SB (0.197) and SC (0.190). The analysis of the above two aspects showed that SA had the highest genetic diversity, followed by SD, while SB and SC had the lowest genetic diversity.

**Table 3:** Nucleotide polymorphisms (π) and the polymorphic information content (PIC) of sub-populations at different breeding stages in the HR.

Sub	Year of release	Accessions	Π (× 10^–3^)	PIC
A	1963–1980	12	1.54	0.242
B	1983–1988	8	1.27	0.197
C	1991–2000	7	1.24	0.190
D	2001–2010	90	1.41	0.234
E	2011–2017	64	1.38	0.227

*Sub*. Sub-population names of different breeding stages in HR, *Accessions* Accession numbers of every sub-population, *π* Genetic diversity (× 10^–3^) of every sub-population, *PIC* polymorphic information content of every sub-population.

## Discussion

Compared with the reference genome (Gmax_275_Wm82.a2.v1), many genetic changes were found in the germplasm resources of HR due to the occurrence of variations. Perhaps it was precisely because of the existence of these mutations that the function of the affected genes were enhanced or weakened or even lost its function. Except for the genes we mentioned in the second part of the results that may be affected by the putative variations, in fact, we also selected 18 genes in soybean that have been functionally verified in previous studies to study the mutations in these genes (Supplementary Table 4). These genes mainly participated in the photoperiod, seed weight, seed shape, pod habits and fatty acid synthesis of soybean. Most of the variants in these genes were located in introns, 3'UTR regions and 5'UTR regions, but still some variants occurred in exons of these genes, and may lead to changes in protein coding products. We detected 1, 3, 1, 1, 1, 2, 3 "missense_variant" type variants in *Glyma.02G171600*^30^, *Glyma.04G050200*^31^ (J(E6)), *Glyma.06G207800*^32^ (E1), *Glyma.10G221500*^33^ (E2), *Glyma.16G151000*^34^ (GmFT2b), *Glyma.17G036300*^35^ and *Glyma.19G194300*^36^ (Dt1), respectively. These variants were likely to lead to changes in the products encoded by these genes. Notably, a "stop_gained" type variation was detected in *Glyma.10G221500* and the results of snpEff annotation showed that this variation had a "HIGH" effect on *Glyma.10G221500*, and this gene is a famous soybean photoperiod related gene, which was named as *E2*. What is well known to us all is that soybean is very sensitive to changes in photoperiod and it is a typical short-day plant. Photoperiod affects the latitude adaptability and yield of soybean largely. The above findings will help us to understand the photoperiod adaptation mechanism of soybean in HR and provide genetic resources for soybean molecular breeding. In the near future, more work needs to be done to verify these putative variants affected genes, once the authenticity of the genetic effect of the variations were determined, these variations will guide us to understand the adaptation mechanism of plants to the environmental conditions in HR.

PCA, NJ tree and structure analysis all just told one message that there was no obvious trend of grouping in the only soybean cultivars constructed population in HR. Through pedigree analysis by previous studies, it was found that most of the soybean cultivars in HR were related to each other, and a considerable number of soybean cultivars had common ancestral parent consanguinity^9^. As a result, the genetic basis of germplasm in HR was relatively narrow, the genetic similarity among cultivars was relatively high, and in hence, it was difficult to have an obvious clustering phenomenon. In addition, in this study, a population consisted only of cultivated varieties had a relatively narrow genetic variation panel when comparing with a population that composed by wild, landrace and cultivated varieties, and cultivars in the same location were bred from a few ancestral parents, leading to a very similar genetic base. Furthermore, comparing with the domesticated varieties from wild materials, the cultivated population selected had a relatively short breeding time span, so that the varieties in different breeding periods did not have enough time to accumulate enough variations to produce significant differences, which, in hence, again resulted in a very similar genetic content between different cultivars in HR population. Bruce et al*.*^28^ got a similar result in his study and proved that a single type population indeed had a very narrow genetic basis.

SA had the longest breeding time, due to the frequent recombination in the genome, SA exhibited the highest genetic diversity (π and PIC). However, in this series of changes, the genetic diversity reached the lowest peak in SC (π = 1.24 × 10^–3^, PIC = 0.190) and then displayed an obviously increase in SD (π = 1.41 × 10^–3^, PIC = 0.234) and later in SE the genetic diversity showed a slightly changing. Why was there an inflection point between SC and SD? It was speculated that the varieties bred in HR region might have experienced the most stringent artificial selection during this period^25,37^, which leads to a violent decrease in genetic diversity. Yet, after the period of SC, some breeders gradually realized that only relying on local varieties for variety improvement was not cost-effective and in breeding, we should pay attention to the diversity of genetic resources, make full use of various types, broaden the genetic basis, select parents with distant genetic relationship, and make full use of germplasm resources at home and abroad^9^. As a result, a large number of exotic varieties were introduced, and new variants were entered^38,39^. At the meantime, some breeders have discovered a wealth of excellent allelic variants contained in wild materials and further used them as breeding parents in the subsequent breeding work, which resulting in a high genetic diversity in SD. Moreover, there existed another latent factor that should be considered, which was the small number of samples in SC. There were only seven cultivars in SC, and it is possible that this number of cultivars was not enough to represent the genomic information of this breeding period, and further resulted in false positive in calculations of some statistics. In the future, we will try our best to collect more representative varieties to improve the relevant research content involved in this study.

The changing pattern of genetic diversity was not consistent with the conclusion obtained by previous studies using national or regional representative samples, including wild, landrace and cultivated varieties^22,40^, indicating the necessity of the present study. Thanks to the unremitting efforts of breeders, the genetic diversity of the bred cultivars in the HR had been maintained although there had been different trends in history, which implied the development of different breeding work in different breeding periods. Next, on the basis of ensuring the existing genetic diversity in the HR, breeders seem to be able to focus on the existing wild materials or excellent foreign germplasm resources to identify new alleles for important soybean traits, and thus further improve the diversity of cultivars and the performance of critical traits. In current study, an attempt was made to analyze the genetic structure of the bred varieties population in HR, hoping to provide ideas and references for future research.

## Materials and methods

### Plant materials and DNA extraction

181 soybean varieties were selected from the main families of soybean cultivars bred in the Huang-Huai-Hai region (HR). According to the breeding time, they were divided into five sub-populations: SA (1963–1980), SB (1983–1988), SC (1991–2000), SD (2001–2011) and SE (2011–2017), and the five sub-populations contain 12, 8, 7, 90 and 64 accessions respectively. Notably, among the 12 varieties in SA, there are 2 ancestral parents and 10 cultivars that were bred in the year from 1963 to 1980 (Supplementary Table 5). The soybean accessions were planted at Dangtu Experimental Station of Nanjing Agricultural University, Nanjing, China in 2019 for the utilization for the present study.

Following to the hexadecyltrimethylammonium bromide (CTAB) method^41^, fresh leaf tissues were collected from field-grown plants, which were at V4–V5 vegetative stage, with a single plant sampled per genotype, representative of the whole plot to avoid off-types within the plots. Then fresh leaf tissues were quickly sampled and kept in liquid nitrogen and further for DNA extraction. The OD value (260 nm/280 nm) of the extracted DNA sample was determined by spectrophotometer, and the DNA was examined by 1% agarose gel electrophoresis to ensure that the extracted DNA was not degraded or contaminated by impurities. Still further, common concentration and purity of DNA were detected by Nanodrop, and the concentration of DNA was accurately quantified by Qubit fluorescence. It was required that the total amount of genomic DNA of non-pollution and non-degradation DNA should be more than 10 μg for sequencing.

### Sequencing, variation detection and imputation

The WGS-Seq (Whole Genome Resequencing) was done for variant genotyping for all the 181 selected cultivars in the present study, which was conducted at SHBIO, Shanghai, China. With the help of the Illumina Hiseq2500 sequencing platform and combined with the paired-end sequencing method, the sample genome resequencing analysis was carried out. BWA-MEM^42^ (version 0.7.12) was used to map the preprocessed paired-end reads onto the reference soybean genome Williams82^43^ (Gmax_275_Wm82.a2.v1) with a -M added to mark shorter split hits as secondary and other parameters used default values. The original “.fastq” files were converted into “.bam” files by samtools^44^, and the Markduplicates tool of Picard^45^ (version:1.87) was used to mark possible PCR duplicates, and the flagstat tool of samtools^45^ was used to compile statistics of mapping information.

Mutation detection was done by employing the HaplotypeCaller and GenotypeGVCFs module of the GATK^46^ (version 4.1.1.0). The minimum read mapping quality was set to 20, and the default parameter settings were selected for the rest. First, we used GATK to perform a mutation detection on the data after mapping, sorting, and marking duplications to obtain a VCF file containing the mutation set. Second, we treated the first step obtained variant data as a known mutation set, and adopted the BaseRecalibrator and ApplyBQSR module to re-calibrate the sequencing bases’ quality of all reads in the “.bam” file, and utilized the newly generated bam file together with the HaplotypeCaller and GenotypeGVCFs modules of GATK to generate a second mutation detection result. Next, we made use of GATK's VariantFiltration module for quality control for the second mutation detection result, and all the quality control parameters were the default settings of the software. Finally, a VCF file containing the original SNPs and InDels were ready for the next step analysis.

After all the procedures above, A total of 11,624,289 SNPs and 2,520,208 InDels (small insertions and deletions < 50 bp) were identified from the analyses of the genomes of 181 cultivars in HR. Two subsets of all SNPs detected were defined using the following filtering criteria: (1) a simply filtered data set containing 11,185,589 SNPs by removing SNPs, which was monomorphic or having more than two alleles; (2) a core SNP set containing 4,666,538 (Table 4) high quality SNPs according to the criteria of missing and heterozygosity rate ≤ 10% and minor allele frequency (MAF) ≥ 2%^47^.

**Table 4:** Distribution of SNPs of the core SNPs set on every chromosome.

Chromosome	SNPs	Chromosome	SNPs
Chr01	230,431	Chr12	125,812
Chr02	211,263	Chr13	237,867
Chr03	273,640	Chr14	209,270
Chr04	277,013	Chr15	368,802
Chr05	138,715	Chr16	260,243
Chr06	273,489	Chr17	220,101
Chr07	191,174	Chr18	409,735
Chr08	207,390	Chr19	230,387
Chr09	250,745	Chr20	185,048
Chr10	228,476	Total	4,666,538
Chr11	136,937		

The untyped genotype data of the core SNP set were then imputed by beagle^48^ software with a sliding window of 10,000 and a step length of 1000.

### Annotation of SNPs and InDels

SNP and InDel annotation were both performed according to the soybean reference genome (Gmax_275_Wm82.a2.v1) using snpEff^49^ (Version: 5.0). The genome region classification of SNPs and InDels in the present study was consistent with that in Zhou^22^, with just one point in difference, which was that the present study defined a 5 kb interval from the start or stop codon sites as an upstream or downstream variation. The simply filtered SNP set and all the InDels were used for the annotation analysis, and for the following analysis below, they were all basing on the core SNP data set. There existed one more thing needing to be made clear, which was that in the present study we refer to a variant site from the simply filtered SNP set, of which the missing rate was less than 10%, and all the rest accessions showing a mutation as the site that mutations had occurred in all samples.

### Population structure, clustering and LD decay analysis

We conducted the principal component analysis (PCA) using Plink^50^. The neighbor-joining tree was constructed using MEGA^51^ (version: 7.0) with neighbor-joining algorithm^52^, and the bootstrap value was set to 1000. Terastructure^53^ basing on the machine learning algorithm was used to estimate the structure of population in the present study. The range of k value was set to 2–20, and the calculation of each K value was repeated for three times. Then, we extract the final validation likelihood for each run and averaged overall reps and drawn the averaged values into a line chart, and finally, we chose the value of K where the validation likelihood plateaus. We used Plink^50^ to compute the degree of linkage disequilibrium (LD) of each sub-population. We set 1 Mb as the window length to calculate the LD value between SNP pairs, the LD of SNP pairs between different chromosomes were ignored in this study, and finally, we used the in-house R scripts to visualize the LD decay trend.

### Genetic diversity analysis

Nucleotide diversity^54^ (π) and polymorphic information content (PIC)^55^ were both used to describe population genetic diversity. Nucleotide diversity refers to the average value of nucleotide difference at each site between any two nucleotide sequences in a population, which can be computed as $\pi { = }\sum\nolimits_{ij} {x_{i} } x_{j} \pi_{ij}$, where *x*_*i*_ is the frequency of sequence *i*, *x*_*j*_ is the frequency of sequence *j*, and π_*ij*_ is the number of nucleotide differences between sequence *i* and *j*. Polymorphic information content (PIC) is a measure of the amount of information that can be provided by the polymorphism of a genetic marker in linkage analysis. Now it was often used to measure the degree of locus polymorphism, which can be computed as $PIC_{l} = 1 - \sum\nolimits_{u} {p_{lu}^{2} } - \sum\nolimits_{u} {} \sum\nolimits_{v,v > u} {2p_{lu}^{2} } p_{lv}^{2}$, where *P*_*lu*_ is the frequency of the *uth* allele of the *l* marker and *P*_*lv*_ is the frequency of the *vth* allele of the *l* marker, and *v* is bigger than *u* in number. π was calculated by -window-pi command of vcftools-0.1.15^56^ software with 100 kb (no overlap between windows) as the calculation window and a step length of 10 kb, π of sub-populations were the average of results from all calculation windows on the genome. According to its definition, PIC was calculated by a self-written Python (version: 3.7.1) script, and the average PIC of all loci was the population PIC value.

### Research involving plants

The use of plants in the present study complies with international, national and/or institutional guidelines.

### Ethical standards

Experimental research on the plants and the writing process of this manuscript comply with the current laws of China.

## Supplementary Information

Supplementary Information.
