# Supplementary material for: Putative variants, genetic diversity and population structure among Soybean cultivars bred at different ages in Huang-Huai-Hai region
Source: Sci Rep. 2022 Feb 11;12:2372. doi: 10.1038/s41598-022-06447-6 (PMC8837640; doi:10.1038/s41598-022-06447-6)
Supplement: Supplementary file 1 — Supplementary Information. [file 41598_2022_6447_MOESM1_ESM.pdf]

Supplementary Material

Supplementary Tables

Supplementary Table 1 Sequencing information for the Huang-Huai-Hai cultivated soybean accessions

Accession	Total reads	Clean reads	Mapped reads	Mapped ratio (%)	Genome coverage (%)	Mean depth
E003	97624138	96495452	92849854	96.22	95.47	12.57
E004	75225288	74324008	73485714	98.87	95.48	10.18
E005	79433042	78411504	77774714	99.19	95.38	10.71
E006	76929864	76183998	75115017	98.6	95.37	9.51
E007	85617332	84764048	78722339	92.87	95.6	9.95
E009	75560264	74573824	69130824	92.7	95.04	9.36
E011	67133138	66664902	66052834	99.08	95.02	8.48
E012	71759084	70763580	68376359	96.63	95.43	9.41
E014	76717854	75799666	68988167	91.01	95.4	9.26
E018	84975200	84065764	82257819	97.85	95.55	11.23
E019	67088636	66457226	65055847	97.89	95.03	8.35
E020	64958662	64106612	62772809	97.92	95.2	8.71
E021	72328500	71483496	68561710	95.91	95.13	9.4
E022	70602540	69764680	65613190	94.05	95.91	9.01
E023	85012584	84061454	83453882	99.28	95.66	11.38
E024	74613256	73789942	61344875	83.13	95.39	8.18
E025	67594744	66459858	57234129	86.12	95.39	7.69
E027	65247720	64549670	64070885	99.26	95.14	8.87
E028	63038006	62392854	61653653	98.82	95.04	8.51
E029	78237386	77590280	74500668	96.02	95.48	9.26
E030	80291278	79388904	76129409	95.89	95.44	10.47
E031	99875070	98643240	95652789	96.97	95.59	13.44
E032	80615234	79746260	78364711	98.27	95.59	10.75
E037	76852772	76019182	73170611	96.25	95.25	10.11
E039	71261858	70774154	67438385	95.29	95.26	8.57
E042	84997632	84029386	77244045	91.93	95.75	10.3
E043	92703600	91906618	89017029	96.86	96.41	11.35
E044	75450744	74405454	67549569	90.79	95.61	9.23
E045	78000450	77350820	74848096	96.76	95.54	9.53
E046	89944400	88838672	84474047	95.09	95.63	11.5
E047	85308248	84539536	78460095	92.81	95.69	9.7
E051	79428042	78798692	75294384	95.55	95.78	9.55
E052	85679254	84572882	73440584	86.84	95.84	9.65
E053	73904578	72819022	54582771	74.96	95.19	6.97
E054	70488206	69668106	61123154	87.73	95.17	7.74
E059	69012900	68456506	65446514	95.6	96	8.28
E060	84981846	84048942	80009153	95.19	95.63	10.69
E062	80913372	80116782	70703324	88.25	96.15	8.73
E065	62927498	62099266	50985161	82.1	94.63	6.64
E069	76741892	76057284	63974551	84.11	95.48	7.9
E073	79294174	78309260	64858598	82.82	95.66	8.38
E075	117488832	116263044	112940587	97.14	96	14.73
E076	78748892	77661680	63225489	81.41	95.21	8.14
E077	68722722	67765868	60104057	88.69	96.05	7.96
E081	82957582	81971324	76016376	92.74	95.48	10.29
E085	97113146	95888110	83336136	86.91	95.68	10.81
E088	77003712	75854770	61276615	80.78	95.44	7.9
E089	78672310	77666702	71671404	92.28	95.61	9.8
E090	66378066	65379758	57924423	88.6	95.53	7.76
E091	69843864	68977190	58803544	85.25	95.15	7.66
E092	109911598	108866004	99314164	91.23	96.11	12.52
E093	79956272	78799434	68883152	87.42	95.71	9.17
E094	88947286	87960916	86422247	98.25	96.27	11.67
E095	71052440	70125266	69425792	99	95.23	9.52
E096	95414272	94455056	89657122	94.92	95.44	11.35
E097	73159484	72166370	62035961	85.96	95.01	8.38
E098	78333338	77140908	69759254	90.43	96.39	9.46
E099	84101192	83343430	80823490	96.98	95.53	10.3

E100	77666452	76733178	72438970	94.4	95.48	9.89
E102	68012372	67268782	64329614	95.63	95.23	8.88
E103	76402234	75229496	61704462	82.02	95.19	8.16
E104	82357420	81386098	74153988	91.11	95.68	9.89
E105	75018080	74162620	59736130	80.55	95.59	7.76
E106	74257940	73380182	70872830	96.58	95.37	9.67
E110	83483658	82544764	79335326	96.11	95.3	10.86
E112	82498758	81662970	79708794	97.61	95.66	10.13
E113	79578860	78816012	69336988	87.97	94.99	8.65
E115	92492892	91533340	85334465	93.23	95.58	11.06
E117	85496168	84297320	81603059	96.8	95.56	11.26
E118	71757570	70940036	67161556	94.67	95.35	9.13
E119	78714024	77982174	75970279	97.42	95.18	9.75
E120	123945338	122491714	118741314	96.94	95.77	15.82
E121	86039342	85210512	81557170	95.71	95.46	10.31
E122	84720818	84010368	82102112	97.73	95.99	10.47
E123	80337048	79447012	77809206	97.94	95.99	10.08
E124	71930586	71380580	70143132	98.27	95.24	8.84
E129	77124980	76516598	75975623	99.29	95.07	9.68
E130	103568796	102018256	96547780	94.64	95.65	12.91
E131	122551004	121251442	115537419	95.29	95.71	15.44
E137	84485936	83771016	82468632	98.45	95.18	10.4
E138	76984378	76310092	75162229	98.5	95.02	9.56
E139	82484550	81507332	78215970	95.96	95.39	10.65
E140	98325864	97344274	91463726	93.96	95.52	11.39
E141	85985328	85235706	84071813	98.63	95.53	10.68
E142	79040060	78400390	76422156	97.48	95.34	9.66
E143	91049686	90045930	88784309	98.6	95.64	11.33
E144	92452890	91560188	86636921	94.62	95.36	11.55
E155	73672082	73012380	71623401	98.1	95.63	9
E156	101735704	100525308	96580229	96.08	95.54	12.88
E157	103617440	102648696	99172788	96.61	95.67	12.16
E158	101886358	100697718	91178140	90.55	95.69	12.02
E161	88163980	87054730	79131922	90.9	95.74	9.87
E163	76136360	75276146	72635859	96.49	95.87	9.86
E164	74401222	73587410	72074378	97.94	95.64	9.9
E165	76996748	76009216	74070424	97.45	95.76	10.12
E166	70668004	69804798	61708524	88.4	95.33	8.15
E167	76848142	75768814	66971830	88.39	95.83	8.86
E169	76343044	75785278	72322183	95.43	95.77	9.2
E170	66351854	65676284	64796832	98.66	95.47	8.93
E172	111340518	110207428	103552705	93.96	97.08	13.27
E173	76925512	75924824	73101998	96.28	96.93	9.91
E174	72403312	71597178	70862417	98.97	95.59	9.74
E175	126049540	124918800	123983055	99.25	95.68	16.11
E176	122039616	120819304	110845261	91.74	96.14	13.98
E177	101684604	100598122	99530224	98.94	95.51	13.03
E178	129361936	128090430	125465993	97.95	95.48	16.04
E179	76825064	75900406	75274419	99.18	95.07	10.33
E180	89260974	88152418	87577902	99.35	95.98	12.06
E181	79441570	78429782	70546509	89.95	95.66	9.33
E182	110265166	109238440	108202774	99.05	95.38	13.85
E184	81172912	80164474	79169157	98.76	95.7	10.83
E185	127830522	126585436	124622225	98.45	96.03	16.22
E186	120676856	119489156	116692297	97.66	95.84	15.18
E188	71899964	71188046	67492961	94.81	95.33	9.15
E189	79127500	78126154	66475882	85.09	95.39	8.77
E190	76784030	75719792	71945143	95.01	96.04	9.81
E191	68366390	67783346	63511880	93.7	95.72	8.08
E192	81965954	81238778	75281980	92.67	95.44	9.43
E193	69452376	68717212	65771606	95.71	95.25	8.94
E194	81822108	80947790	75318895	93.05	95.41	9.55
E195	79776254	79210696	78444285	99.03	95.13	10.07
E196	73829680	73298630	71612566	97.7	95.53	9.1
E197	70279712	69478294	64053229	92.19	95.49	8.63
E198	122211726	121171674	117863196	97.27	95.86	15.23
E199	126047672	124897268	120982909	96.87	95.98	15.65
E200	86948898	86039258	85377602	99.23	95.59	10.88
E201	68962270	68251802	67734899	99.24	95.35	9.29

E202	107749550	106836964	102106135	95.57	95.91	13.49
E203	83920546	82946058	80905142	97.54	95.5	10.37
E204	71435502	70631804	68077463	96.38	95.87	9.35
E205	70693124	69782958	66266841	94.96	95.63	9.04
E206	75715850	74806610	71124008	95.08	95.34	9.7
E207	73355552	72530752	65461264	90.25	95.55	8.78
E209	107559222	106569320	103717503	97.32	95.89	13.86
E213	78762884	77941762	77534983	99.48	95.46	10.66
E214	71548270	70747322	70049074	99.01	95.38	9.63
E215	78495064	77541528	70950567	91.5	96.28	9.53
E216	118439302	117354364	108148042	92.16	95.5	13.91
E220	104927116	103909924	101420750	97.6	95.52	13.18
E222	83188462	82332876	81133439	98.54	95.4	11.2
E236	77074036	76179034	75148959	98.65	95.55	10.33
E242	66665726	65856740	63797711	96.87	95.46	8.71
E244	108114904	107146970	103572882	96.66	96.16	13.51
E245	82941556	81959014	78578977	95.88	95.86	10.71
E246	91127748	90087820	85298209	94.68	95.92	11.08
E247	73065738	72522812	71629957	98.77	95.28	9.14
E248	90456430	89573324	89021157	99.38	95.74	12.27
E249	106906658	105935062	104976468	99.1	96.15	13.74
E250	80687968	79835438	76528932	95.86	95.28	9.79
E251	92346398	91562312	90785131	99.15	95.83	11.41
E252	67032654	66460100	66316102	99.78	94.49	8.39
E253	71116680	70392186	69929400	99.34	94.81	9.03
E254	78075742	77473258	77183846	99.63	95.27	9.96
E255	84171228	83386440	82816118	99.32	95.69	10.53
E256	79330046	78584174	77990466	99.24	95.53	9.99
E257	86661058	85762110	76142555	88.78	95.05	9.35
E258	77094014	76403050	76065858	99.56	95.28	9.71
E259	103659856	102673030	95199769	92.72	95.68	11.65
E260	78352678	77483740	73884804	95.36	95.09	9.47
E261	71872134	71340830	70828846	99.28	95.54	9.06
E262	96629610	95706312	94722858	98.97	95.74	12.06
E263	94654374	93789424	92986007	99.14	95.73	11.65
E264	89494172	88513266	74802707	84.51	95.83	9.35
E265	102481486	101664766	101106967	99.45	96.17	12.65
E266	76447844	75787730	73719943	97.27	95.67	9.39
E267	75377094	74574228	63805376	85.56	95.64	7.99
E268	85581606	84814768	78645775	92.73	95.36	9.89
E269	85739510	85010884	83645802	98.39	95.59	10.69
E270	102935674	101989384	101367570	99.39	95.79	12.68
E271	81303346	80474270	79675281	99.01	95.73	10.16
E272	88640972	87741350	87205857	99.39	95.72	11.05
E273	77091304	76494704	72568011	94.87	94.93	9.23
E274	79366490	78772022	76897002	97.62	95.53	9.8
E275	85800102	85070738	83997252	98.74	95.46	10.54
E276	76953592	76156120	75476657	99.11	95.34	9.64
E277	90583222	89786542	89320006	99.48	95.65	11.23
E278	80521876	79505080	74647304	93.89	95.42	9.5
E279	77912280	77203670	76507013	99.1	95.21	9.85
E280	92785252	91906746	91318712	99.36	95.67	12.47
E281	97596156	96685814	88749241	91.79	95.62	11.19
E282	80555090	79578772	79050077	99.34	82.13	10.21

Supplementary Table 2 Variation information on every chromosomes for the Huang-Huai-Hai cultivated soybean accessions

Chromosome	Length (bp)	SNPs	InDels	Density of SNPs (SNPs/Mb)	Density of InDels (InDels/Mb)
Chr01	56831624	621769	120914	10940.55	2127.58
Chr02	48577505	489717	117321	10081.15	2415.13
Chr03	45779781	676692	153956	14781.46	3362.97
Chr04	52389146	605623	121938	11560.09	2327.54
Chr05	42234498	386990	93121	9162.89	2204.86
Chr06	51416486	677539	153827	13177.47	2991.78
Chr07	44630646	463470	120144	10384.57	2691.96
Chr08	47837940	460427	120593	9624.72	2520.87
Chr09	50189764	556800	126331	11093.90	2517.07
Chr10	51566898	548192	120186	10630.70	2330.68
Chr11	34766867	301268	75118	8665.38	2160.62
Chr12	40091314	335297	82493	8363.33	2057.63
Chr13	45874162	517284	142790	11276.15	3112.65
Chr14	49042192	543305	118857	11078.32	2423.57
Chr15	51756343	824461	171126	15929.66	3306.38
Chr16	37887014	601443	147801	15874.65	3901.10
Chr17	41641366	467590	107545	11228.98	2582.65
Chr18	58018742	998064	214420	17202.44	3695.70
Chr19	50746916	578905	116736	11407.69	2300.36
Chr20	47904181	530753	94991	11079.47	1982.94
Total	949183385	11185589	2520208	11784.43	2655.13

Length: The chromosome length of Wm82.a2.v1 and the unit is bp.

Supplementary Table 3 Whole-genome SNP and InDel distribution of every population

Type	Pop.	SUM	Exonic	Intergenic	Intronic	Splicing	Upstream	Downstream	3'UTR	5'UTR
SNP	Whole	11185589	370289	6136859	466503	23806	2548627	1466744	105080	67681
	A	7340332	249782	3774807	328368	16747	1819942	1025557	76790	48339
	B	5708103	203405	2872780	262999	13544	1445430	807793	61963	40189
	C	5179593	181386	2618986	239406	12335	1304783	731505	55674	35518
	D	9844109	325515	5388525	412294	20873	2251900	1292886	92696	59420
	E	8802694	292086	4809450	371268	18785	2015961	1159002	82976	53166
InDel	Whole	2520208	47724	975395	146578	7468	844916	427748	38786	31593
	A	1690598	28537	603256	100301	4854	604456	299264	27838	22092
	B	1359082	23579	474431	82494	4034	491271	241838	22816	18619
	C	1257602	21819	442609	76160	3739	451878	223599	20961	16837
	D	2236687	40981	857411	130879	6484	756492	381423	34765	28252
	E	2042248	36858	780495	120035	5977	692207	348872	31785	26019

Pop.: Abbreviated form of population.

Supplementary Table 4 SNPs in functional genes

Gene model	Gene	Function	SNPs	3V	5V	DV	IV	MV	SRV	SGV	SV	UV
Glyma.02G171600	-	Seed shape	3	0	0	0	2	**1**	0	0	0	0
Glyma.04G050200	J(E6)	Photoperiod	13	0	2	0	2	**3**	0	0	6	0
Glyma.04G156400	E1La	Photoperiod	1	1	0	0	0	0	0	0	0	0
Glyma.05G019200	-	Seed shape	4	0	0	0	0	0	0	0	4	0
Glyma.05G096500	-	Seed shape	1	0	0	0	0	0	0	0	1	0
Glyma.06G207800	E1	Photoperiod	1	0	0	0	0	**1**	0	0	0	0
Glyma.08G363100	E10	Photoperiod	0	0	0	0	0	0	0	0	0	0
Glyma.10G221500	E2	Photoperiod	54	1	0	0	50	**1**	0	1	1	0
Glyma.10G244400	-	Seed weight	6	1	0	0	2	0	0	0	1	1
Glyma.14G194300	FAD3A	fatty acid	4	1	0	0	2	0	0	0	1	0
Glyma.16G044100	GmFT5a	Photoperiod	2	1	0	1	0	0	0	0	0	0
Glyma.16G150700	E9	Photoperiod	26	2	0	14	9	0	1	0	0	0
Glyma.16G151000	GmFT2b	Photoperiod	31	0	0	0	24	**1**	2	0	4	0
Glyma.17G036300	-	Seed shape	6	0	0	0	0	**2**	0	0	4	0
Glyma.17G221100	-	Seed shape	21	2	0	16	0	0	0	0	2	1
Glyma.19G194300	Dt1	pod habit	8	0	0	0	5	**3**	0	0	0	0
Glyma.19G224200	E3	Photoperiod	5	0	1	0	2	0	1	0	1	0
Glyma.20G090000	E4	Photoperiod	0	0	0	0	0	0	0	0	0	0

SNPs: the SNP number in the corresponding gene

3V: 3_prime_UTR_variant

5V: 5_prime_UTR_variant

DV: downstream_gene_variant

IV: intron_variant

MV: missense_variant

SRV: splice_region_variant

SGV: stop_gained

SV: synonymous_variant

UV: upstream_gene_variant

Supplementary Table 5 The basic information of cultivated soybean population in Huang-Huai-Hai region

Accession	Year of release	Province	Sub	Accession	Year of release	Province	Sub	Accession	Year of release	Province	Sub
E003	2008	Anhui	D	E104	2013	Hebei	E	E197	2010	Shandong	D
E004	2013	Anhui	E	E105	2014	Hebei	E	E198	2016	Shandong	E
E005	2006	Anhui	D	E106	2007	Hebei	D	E199	2009	Shandong	D
E006	2009	Anhui	D	E110	2011	Henan	E	E200	2013	Shandong	E
E007	2010	Anhui	D	E112	2006	Henan	D	E201	2014	Shandong	E
E009	2014	Anhui	E	E113	2009	Henan	D	E202	2012	Shandong	E
E011	2007	Anhui	D	E115	2009	Henan	D	E203	2012	Shandong	E
E012	2013	Anhui	E	E117	2007	Henan	D	E204	2016	Shandong	E
E014	2008	Anhui	D	E118	2006	Henan	D	E205	2010	Shandong	D
E018	2008	Anhui	D	E119	2014	Henan	E	E206	2010	Shandong	D
E019	2010	Anhui	D	E120	2009	Henan	D	E207	2014	Shandong	E
E020	2010	Anhui	D	E121	2017	Henan	E	E209	2007	Shanxi1	D
E021	2012	Anhui	E	E122	2013	Henan	E	E213	2010	Shanxi1	D
E022	2012	Anhui	E	E123	2011	Henan	E	E214	2011	Shanxi1	E
E023	2013	Anhui	E	E124	2009	Henan	D	E215	2007	Shanxi1	D
E024	2013	Anhui	E	E129	2006	Henan	D	E216	2011	Shanxi1	E
E025	2015	Anhui	E	E130	2012	Henan	E	E220	2008	Shanxi1	D
E027	2010	Anhui	D	E131	2010	Henan	D	E222	2012	Shanxi1	E
E028	2013	Anhui	E	E137	2007	Henan	D	E236	2014	Shanxi1	E
E029	2012	Anhui	E	E138	2007	Henan	D	E242	2013	Shanxi2	E
E030	2010	Anhui	D	E139	2008	Henan	D	E244	2011	Shanxi2	E
E031	2015	Anhui	E	E140	2011	Henan	E	E245	2012	Shanxi2	E
E032	2013	Anhui	E	E141	2010	Henan	D	E246	2008	Shanxi2	D
E037	2011	Beijing	E	E142	2013	Henan	E	E247	ancestor	Shandong	A
E039	2009	Beijing	D	E143	2013	Henan	E	E248	ancestor	Shandong	A
E042	2010	Beijing	D	E144	2015	Henan	E	E249	1977	Sichuan	A
E043	2007	Beijing	D	E155	2011	Jiangsu	E	E250	1983	Beijing	B
E044	2009	Beijing	D	E156	2014	Jiangsu	E	E251	1985	Henan	B
E045	2007	Beijing	D	E157	2007	Jiangsu	D	E252	1988	Henan	B
E046	2006	Beijing	D	E158	2008	Jiangsu	D	E253	1978	Henan	A
E047	2009	Beijing	D	E161	2009	Jiangsu	D	E254	1971	Shandong	A
E051	2009	Beijing	D	E163	2006	Jiangsu	D	E255	2002	Henan	D
E052	2009	Beijing	D	E164	2012	Jiangsu	E	E256	1985	Jiangsu	B
E053	2010	Beijing	D	E165	2009	Jiangsu	D	E257	1978	Jiangsu	A
E054	2010	Beijing	D	E166	2009	Jiangsu	D	E258	2001	Jiangsu	D
E059	2010	Beijing	D	E167	2011	Jiangsu	E	E259	1963	Shandong	A
E060	2010	Beijing	D	E169	2014	Jiangsu	E	E260	1980	Shandong	A
E062	2006	Beijing	D	E170	2015	Jiangsu	E	E261	1970	Shandong	A
E065	2011	Beijing	E	E172	2014	Shandong	E	E262	1975	Shandong	A
E069	2014	Beijing	E	E173	2006	Shandong	D	E263	2003	Henan	D
E073	2013	Beijing	E	E174	2006	Shandong	D	E264	2001	Hebei	D
E075	2012	Beijing	E	E175	2008	Shandong	D	E265	1985	Henan	B
E076	2014	Beijing	E	E176	2007	Shandong	D	E266	2003	Shandong	D
E077	2015	Beijing	E	E177	2009	Shandong	D	E267	2002	Henan	D
E081	2010	Gansu	D	E178	2010	Shandong	D	E268	1992	Henan	C
E085	2012	Hebei	E	E179	2010	Shandong	D	E269	1997	Henan	C
E088	2008	Hebei	D	E180	2012	Shandong	E	E270	2000	Henan	C
E089	2007	Hebei	D	E181	2005	Shandong	D	E271	2001	Henan	D
E090	2006	Hebei	D	E182	2015	Shandong	E	E272	2003	Henan	D
E091	2007	Hebei	D	E184	2010	Shandong	D	E273	1991	Henan	C
E092	2009	Hebei	D	E185	2013	Shandong	E	E274	1987	Henan	B
E093	2011	Hebei	E	E186	2010	Shandong	D	E275	1971	Henan	A
E094	2006	Hebei	D	E188	2006	Shandong	D	E276	1992	Beijing	C
E095	2008	Hebei	D	E189	2013	Shandong	E	E277	1994	Beijing	C
E096	2008	Hebei	D	E190	2015	Shandong	E	E278	1994	Henan	C
E097	2010	Hebei	D	E191	2014	Shandong	E	E279	1988	Anhui	B
E098	2013	Hebei	E	E192	2006	Shandong	D	E280	2001	Henan	D
E099	2011	Hebei	E	E193	2006	Shandong	D	E281	1975	Henan	A
E100	2007	Hebei	D	E194	2008	Shandong	D	E282	1987	Hubei	B
E102	2009	Hebei	D	E195	2009	Shandong	D				
E103	2011	Hebei	E	E196	2015	Shandong	E				

Sub: the sub-population that the accession belongs.

Supplementary Figures

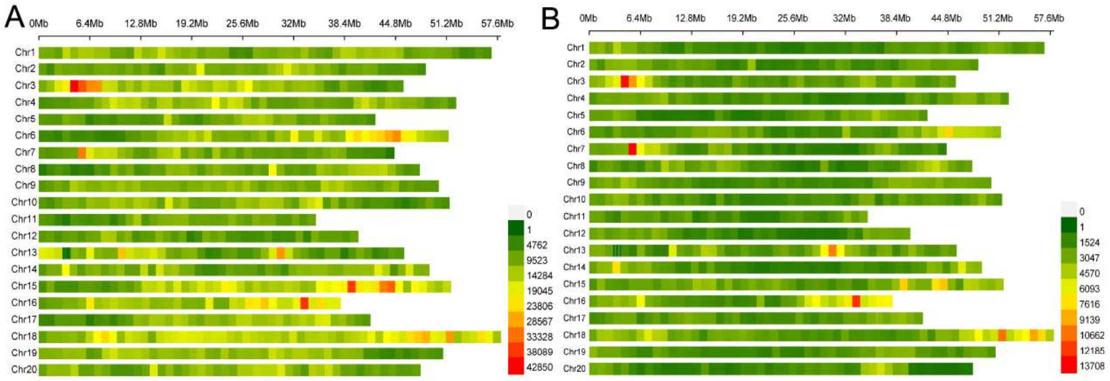

Supplementary Figure 1 SNP and InDel density plot on every chromosome. Note that the plot was generated using CMplot (https://github.com/yinlilin/cmplot).

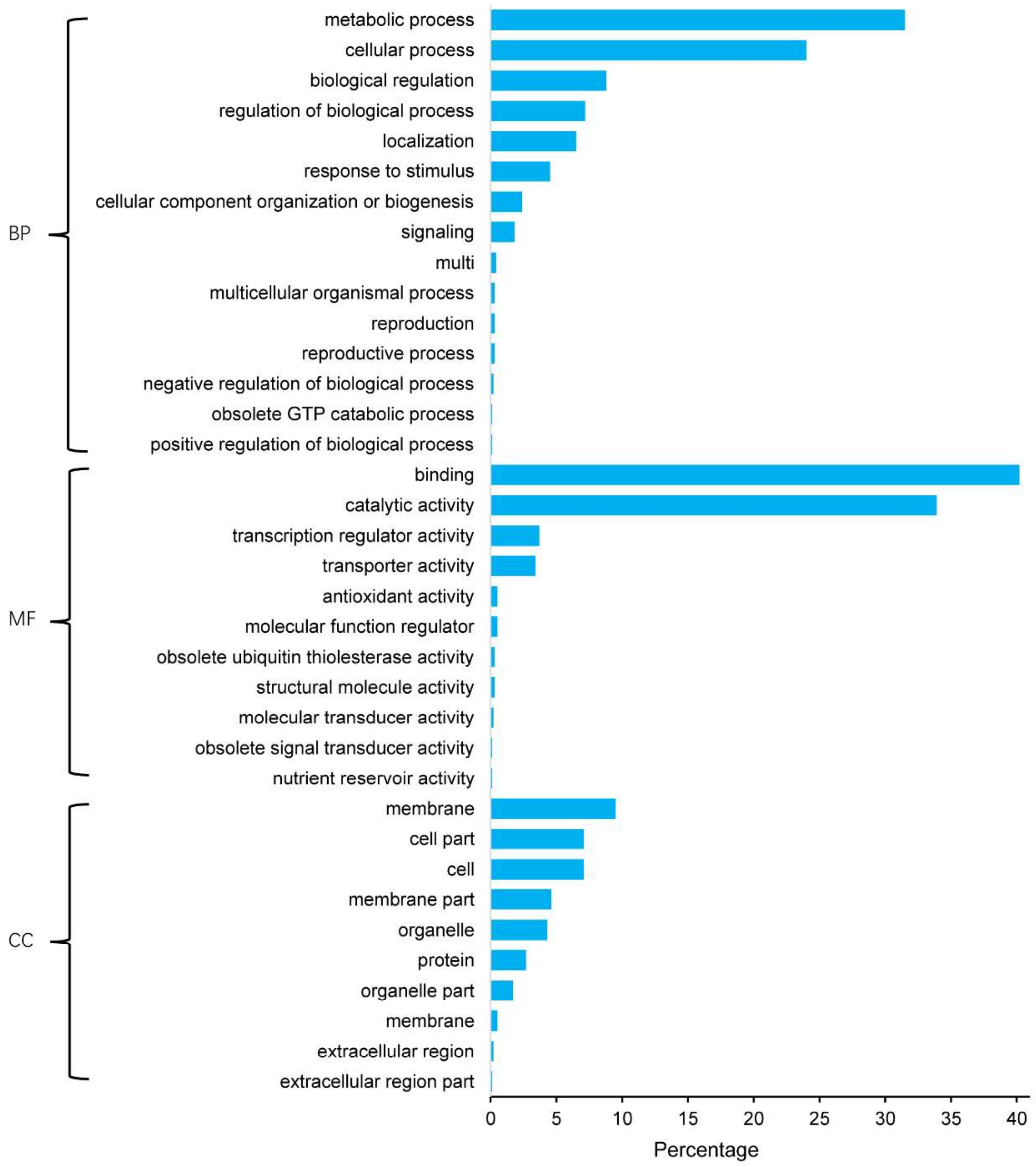

Supplementary Figure 2 Gene ontology annotation plot for genes affected by SNPs, which were mutated in all varieties in HR. The x axis is the percentage of genes under a GO term to the total number of annotated genes. BP: Abbreviation for Biological Process. MF: Abbreviation for Molecular Function. CC: Abbreviation for Cellular Component. Note that the plot was generated using Microsoft Excel 2016.

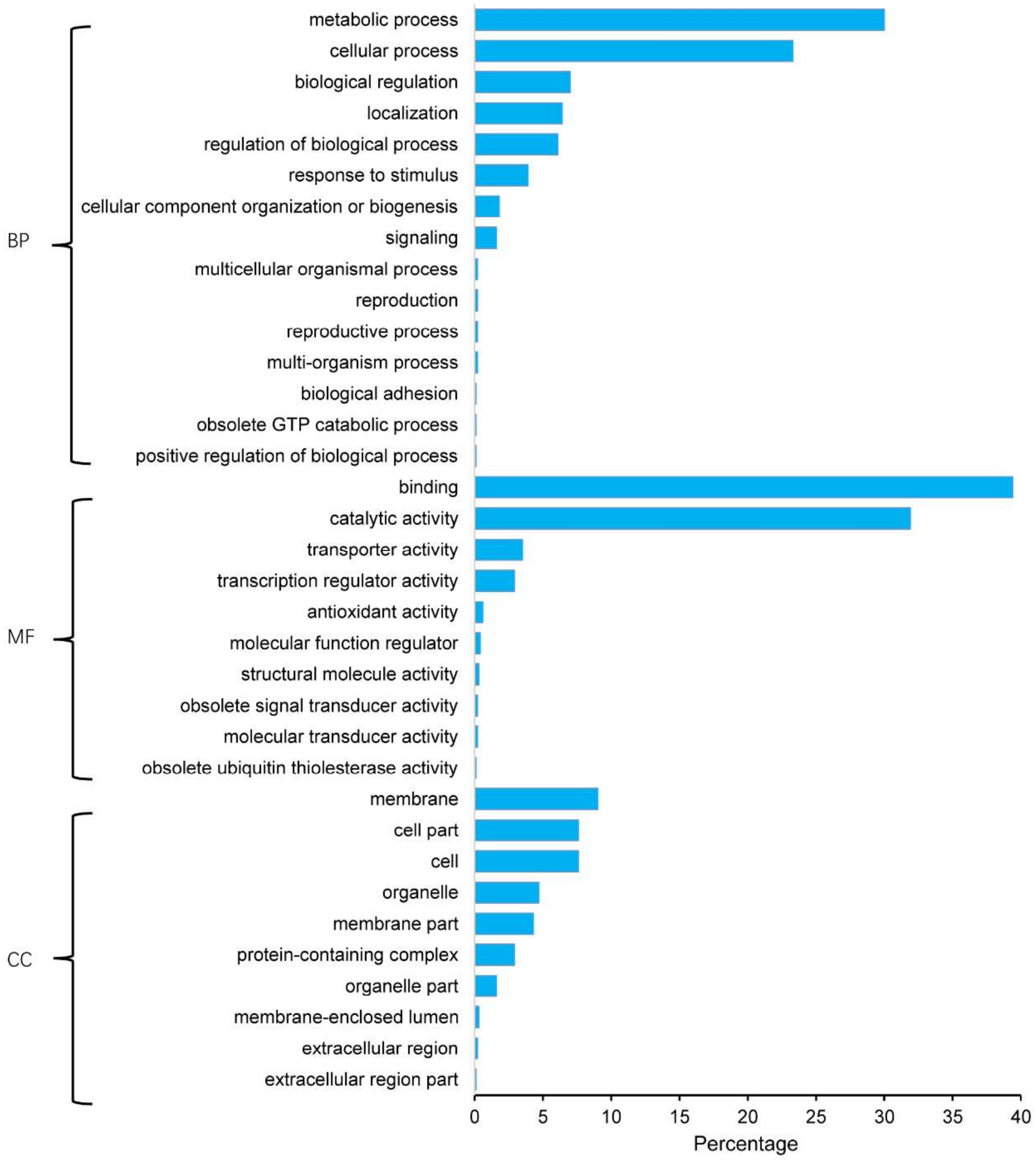

Supplementary Figure 3 Gene ontology annotation plot for genes affected by InDels, which were mutated in all varieties in HR. The x axis is the percentage of genes under a GO term to the total number of annotated genes. BP: Abbreviation for Biological Process. MF: Abbreviation for Molecular Function. CC: Abbreviation for Cellular Component. Note that the plot was generated using Microsoft Excel 2016.

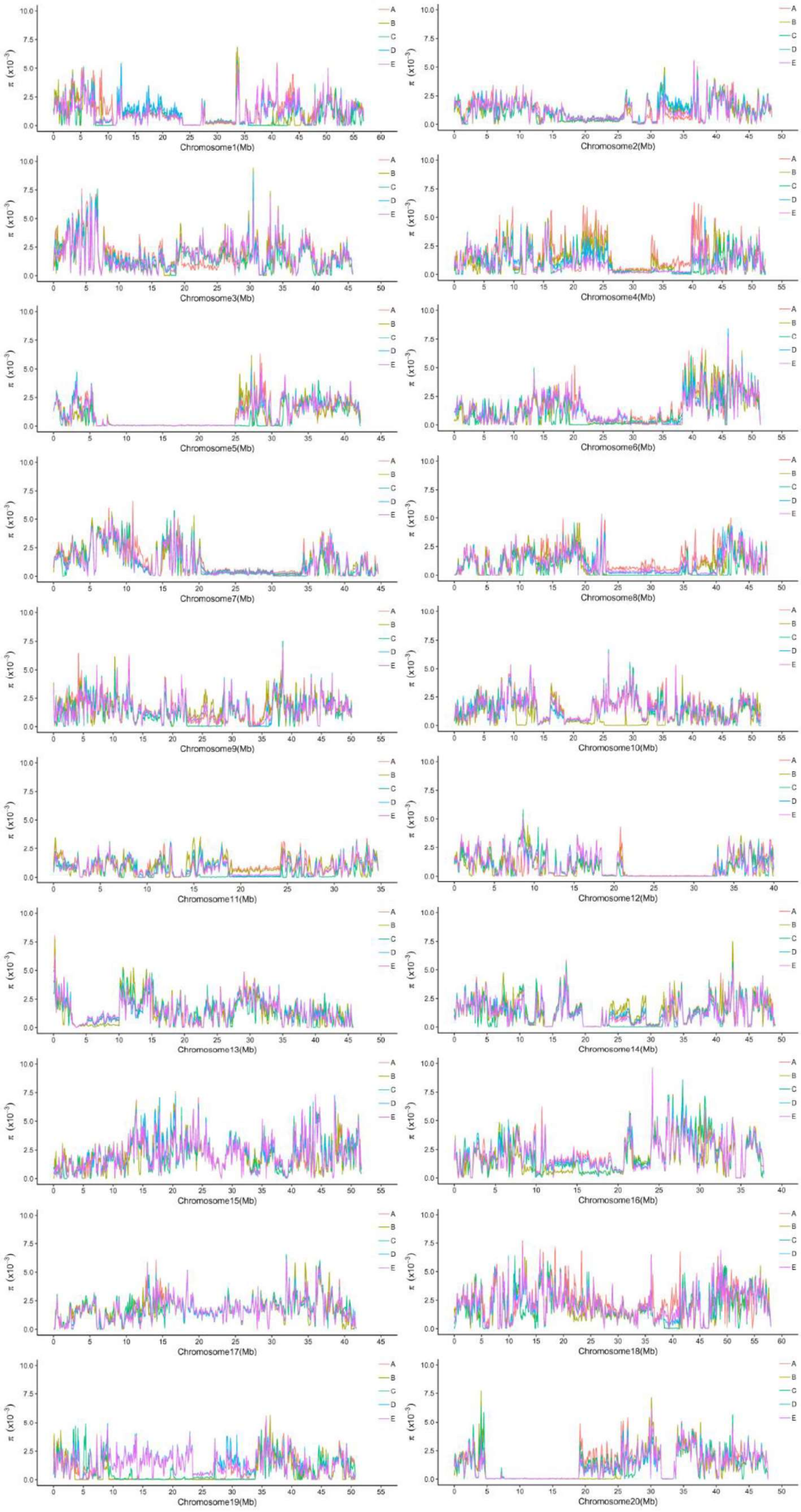

Supplementary Figure 4 Whole-genome genetic diversity (π) of sub-populations. Note that the plot was generated using Microsoft Excel 2016.
